# Association between life-course socioeconomic position and inflammatory biomarkers in older age: a nationally representative cohort study in Taiwan

**DOI:** 10.1186/s12877-017-0598-x

**Published:** 2017-09-02

**Authors:** Yu-Hsuan Lin, Min-Hua Jen, Kuo-Liong Chien

**Affiliations:** 1 Health Promotion Administration, Ministry of Health and Welfare, Taipei, Taiwan; 2 Eli Lilly and the company, Surrey, UK; 3 Institute of Epidemiology and Preventive Medicine, College of Public Health, National Taiwan University, Mailing address: Rm. 517, 5F., No. 17, Xuzhou Rd., Zhongzheng Dist, Taipei City, 100 Taiwan

**Keywords:** Life-course, Socioeconomic position, Inflammation

## Abstract

**Background:**

Evidence of an association between low socioeconomic position (SEP) and inflammatory markers is scant. This study aimed to examine how life-course SEP predicted C-reactive protein (CRP) and interleukin (IL-6) in older age from a national cohort.

**Methods:**

We collected data from 1036 participants in the Social Environment and Biomarkers of Aging Study in Taiwan. Four SEP time points, childhood, young adulthood, active professional life, and older age were measured retrospectively. A group-based trajectory analysis method was used to identify the distinct trajectories of life-course SEP, and trajectory group membership was used as the predictor of CRP and IL-6 levels in older age.

**Results:**

Three trajectories of life-course SEP were identified within the total sample: Low-Low (36.5%), Low-High (26.8%), and High-High (36.7%). Participants in the High-High group had the lowest levels of CRP and IL-6. Compared with those in the Low-Low group, the participants in the Low-High group had a similar adjusted CRP [−0.032 ln mg/L; 95% confidence interval (CI) − 0.193, 0.128] and IL-6 (0.017 ln pg/mL; 95% CI −0.093, 0.128); the participants in the High-High group had a significantly lower level of adjusted CRP concentration (−0.279 ln mg/L; 95% CI: −0.434, −0.125) and similarly lower IL-6 concentration (−0.129 ln pg/mL; 95% CI −0.236, −0.023) .

**Conclusions:**

Life-course SEP is related to the level of CRP and IL-6 in older age. Our data support the notion that life-course SEP predicts inflammatory markers in older age. Low SEP in childhood is related to elevated inflammatory markers in older age. Even after the transition from low SEP in childhood to high SEP in older age, the risk remains. Further study on SEP and inflammation-related disease is warranted.

## Background

The socioeconomic position (SEP) that individuals or groups hold within the structure of society has long and widely been reported as a predictor of cardiovascular disease [1, 2]. The use of inflammatory markers as predictors of cardiovascular disease has been widely studied. Markers of inflammation, in particular C-reactive protein (CRP) and interleukin 6 (IL-6), have been reported as predictors of cardiovascular diseases [3–5]. CRP is a marker of systemic inflammation [3, 6]. IL-6 acts as both a pro-inflammatory and an anti-inflammatory cytokine mediator [7]. Levels of IL-6 and CRP are physiologically linked because of the function of IL-6 in hepatic synthesis and the excretion of CRP [7]. Evidence from observational studies suggests that low SEP is associated with inflammation, atherosclerosis, and the subsequent risk of cardiovascular disease [8–11]. Elevated levels of inflammatory markers were reported for persons of adverse life-course socioeconomic indicators. A systematic review of 25 population-based studies conducted in Western counties revealed inverse associations between CRP levels and SEP. The magnitude of the association was attenuated but remained significant after adjustment for conventional risk factors [12].

SEP has been measured with education, living conditions, occupation, or income respectively in many studies, or with a combination of socioeconomic determinants. These socioeconomic determinants are related to resources as well as exposure and susceptibility to diseases. Health behavior and psychological distress were considered two major pathways by which SEP may influence levels of inflammatory biomarkers. More recent studies have documented the potential links between SEP at multiple points in a life course and its association with the development of cardiovascular diseases [13, 14]. Childhood SEP has been defined frequently according to the parent’s education and occupation, household income, or living conditions in early life. SEP in young adulthood has often been defined according to personal education attainment. Personal employment, income, wealth, or housing conditions were used to defined the SEP time point of active professional life or middle or older age [15]. Frameworks have been conceptualized to illustrate SEP throughout a life course [16–18]. The most frequently tested hypotheses of life-course SEP and health were the accumulation of risk, sensitive (or critical) periods, and social mobility. The accumulation risk model focuses on the total cumulative exposure to SEP throughout a life course, whereas the sensitive or critical period entails the SEP of a certain period in a life course having stronger effects than those of other periods. The social mobility model describes the trajectories of SEP that people are involved in through their life span and is often defined as inter-generational movements of social position from family of origin to adulthood. The pattern of social mobility were found to be significantly associated with health in later life. The health of people who remained stable in an advantaged position is likely to be better when compared to that of those who moved upward from a disadvantaged to an advantaged position. The health of who remained in the disadvantaged position is likely to be worse than that of those who moved upward from a disadvantaged to an advantaged social position [19]. A growing body of literature demonstrates the links between life-course SEP and inflammatory markers among Western populations; however, most studies have attempted to examine the critical period or accumulation hypothesis [20–23]. Controversial or negative findings were reported because of limited time points of SEP measures. For those that examined the social mobility hypothesis, the trajectory of SEP has often been limited to two time points [17, 24]. Although the aforementioned life course frameworks have often been examined as competing theories, the interdependent nature of these frameworks has been suggested and evidence has shown that the extent to which these frameworks are supported between life course SES and cardiovascular risk varies across race/ethnicity [14]. However, few studies have examined the association between SEP and inflammatory markers in Asian populations. Understanding the association between life-course SEP and the level of inflammation may provide insights into the persistent social patterning of cardiovascular risk and prevention. Studies based on representative cohorts and a life-course design, in particular those based on non-Western populations, are warranted to elucidate the links between the SEP that people experience throughout their life course and inflammation later in life. Therefore, the aim of this study was to examine how life-course SEP predicts inflammatory biomarkers in older age. Life-course SEP frameworks, including the “accumulation of risk,” “social mobility,” and “sensitive periods,” were examined to gain a better understanding of the effects of SEP on inflammatory biomarkers.

## Methods

### Study population and protocol

Data were obtained from the Social Environment and Biomarkers of Aging Study (SEBAS), which was an extension of the population-based Taiwan Longitudinal Survey of Aging (TLSA) that began in 1989 [25]. A random subsample of respondents who completed the 1999 TLSA survey were invited to participate in the first wave of SEBAS in 2000. Our analysis focused on the second wave of the SEBAS conducted in 2006, which comprised the surviving participants from SEBAS 2000, aged ≥60 years in 2006, and a randomly selected subset of a younger refresh cohort of the TLSA in 2003, aged 50–57 in 2003 and 53–60 in 2006, resulting in a nationally representative sample of adults aged ≥53 years. The nationally representative sampling design, age composition of the initial and refresh cohorts, and response rates of this prospective cohort have been described previously [26, 27]. The survey protocols of the SEBAS were approved by human subject committees at the Bureau of Health Promotion (Taipei, Taiwan; reformed into the Health Promotion Administration, Ministry of Health and Welfare, Taiwan, in 2013. Official Approval Code: NIFP-IRB-2000-01), Georgetown University (Washington, D.C., USA. Official Approval Code: 1999–195), and Princeton University (Princeton, New Jersey, USA. Official Approval Codes: #1848, #2193, #2791, #3391). It consisted of an in-home face-to-face questionnaire interview, an in-hospital physical examination, and laboratory assays of biomarkers based on a fasting blood specimen. Informed consent was obtained before participation. Sociodemographic variables collected through face-to-face interviews at recruitment of the TLSA were analyzed with the SEBAS and survival data. Data on socio-demographic characteristics (age, sex, and educational attainment), lifestyle (smoking, alcohol drinking, dietary patterns, and exercise), disease at baseline, and family history of stroke and heart-related disease were collected via face-to-face interviews using a structured questionnaire. Anthropometric measurements of weight and height were performed in hospital. Body mass index (BMI) was calculated using body weight in kilograms divided by the square of height in meters. The classification of BMI conformed to the categories determined by the Taiwan Ministry of Health and Welfare. Systolic and diastolic blood pressures were calculated as the average of two seated readings. The two readings were taken 1 min apart by a registered nurse using a mercury sphygmomanometer on the right arm of the individual at least 20 min after the respondent arrived at the hospital. Levels of other biomarkers including total cholesterol, high-density lipoprotein (HDL) cholesterol, and triglycerides were determined at the central laboratory from the fasting blood samples. Low-density lipoprotein (LDL) was estimated indirectly from the concentrations of total cholesterol, HDL cholesterol, and triglycerides.

### SEP throughout a life course

This study takes the life course approach by incorporating exposure in early childhood to adulthood and older age. Variables reflecting SEP at four time points throughout a life course were selected following the theatrical basis and stages proposed by Galobardes [15]. The SEP at the time points was measured according to the father and participant’s education or occupation and a summarized indicator of socioeconomic status. The level of education and major life occupation were collected according to the participant’s self-reported response to the face-to-face questionnaire interview from the baseline survey. The New Occupational Prestige and Socioeconomic Score for Taiwan [28] was applied to categorize the father’s occupation and the participant’s own occupation into groups that ranked from supervisor/executive/professional, technical/semiprofessional, and skilled worker/clerical/sales to unskilled worker and unemployed.

#### Childhood SEP (father’s education and occupation)

Childhood SEP was defined as SEP at 6 years of age and measured according to the father’s education and occupation. The variable was categorized into three groups: low = 0 years, medium = 1–5 years, high = 6 years or more. Occupation was categorized as low = unskilled worker or unemployed, medium = technical/semiprofessional or skilled worker/clerical/sales, and high = executive/professional/supervisor. These education and occupation categorizations were combined into only two or three categories for analyses. The three categories of childhood SEP for descriptive analyses were low (father’s education = low and father’s occupation = low), high (father’ education = high/medium and father’s education = high) and medium (those who were not in low or high categories). These three categories were further grouped into dichotomous categories for trajectory analysis: relatively low (low or medium) and relatively high (high).

#### Young adulthood SEP (own education attainment)

Young adulthood SEP was defined as SEP at age 25, the age at which most Taiwanese people complete their education, and was measured according to personal education attainment. The variable was first categorized into low, medium, and high identically to how the father’s education was categorized. The three categories of young adulthood SEP for descriptive analyses were low = 0 years, medium = 1–5 years, and high = 6 years or more. The dichotomous categories for trajectory analysis were relatively low (low or medium) and relatively high (high).

#### Active professional life SEP (own education attainment and occupation)

Active professional life SEP was defined as SEP at age 40, and measured according to the participant’s own education and major occupation in life. Age 40 was defined arbitrarily because it was the mid-point between 25 and 55 years because age 25 was the age at which most Taiwanese people complete their education, and age 55 is around the average age (55.9) at which Taiwanese people retired [29]. A participant’s own occupation was first categorized into low, medium, and high identically to how the father’s occupation was categorized. The three categories of active professional life SEP for descriptive analyses were low (own education = low and own occupation = low), medium (own education = medium or own occupation = medium), and high (own education = high and own education = high). The three categories were further grouped into dichotomous categories: relatively low (low or medium) and relatively high (high).

#### Older age SEP (summarized indicator of self-related socioeconomic status)

The final point was SEP at the time when the blood samples for inflammatory marker assays were collected. The participant ages ranged from 53 to 96 years. A summarized SEP indicator of self-rated socio-economic status was used to measure SEP at this time point. This instrument, known as the MacArthur Scale of Subjective Social Status, asks respondents to use 10 rungs of a ladder to position themselves socioeconomically relative to other people in their country. This subjective measure has been demonstrated to be a significant predictor of health even in the presence of conventional objective measures of social economic status for older Taiwanese [30]. The response to the question was categorized into low = 0–4 rungs, medium = 5 rungs, high = 6–10 rungs and then further categorized into relatively high = 5–10 rungs and relatively low = 1–4 rungs.

### Inflammatory markers

The inflammatory markers examined in this study were CRP and interleukin (IL-6). All participants were instructed to fast for at least 8 h before their hospital visit. Venous blood samples were drawn and centrifuged on site according to standard operating procedures. High-sensitivity CRP was determined by immunoturbidimetry assay (SIEMENS Healthcare Diagnostics, Tarrytown, NY, USA). The lower limit of detection was 0.012 mg/dL for CRP, and the interassay coefficient of variation (CV) reported by the lab was 2.77%. We measured IL-6 using enzyme-linked immunosorbent assay (R&D Systems, Inc., Minneapolis, MN, USA) with a detection limit of 0.7 pg/mL, and the interassay CV reported by the lab was 12.6%. Aliquots of serum were kept frozen at −70 °C until a batch assay of IL-6 was performed in 2007 and CRP in 2009 in the central lab. Duplicate assays were performed and the average of two readings was computed to reduce variation in the lab results, as reported elsewhere [31]. When the value was reported by the laboratory as below assay sensitivity (BAS) for one or both duplicate assays, we coded the BAS values to the detection limit before calculating the mean between duplicate assays. The outliers were trimmed by recoding them to p75 + 5 × IQR, where p75 indicates the 75th percentile, and IQR is the interquartile range (75th–25th percentile).

### Covariates

Risk factors for cardiovascular disease, including age (53–74; 75–84; and ≥85), gender, ever being diagnosed with hypertension (yes/no), diabetes (yes/no), heart-related disease (yes/no), stroke (yes/no), Framingham risk score (cont.), lifestyle and health behavior, such as body mass index (BMI; underweight, normal, overweight, and obese), smoking (nonsmoker, current smoker, and ex-smoker), alcohol drinking (nondrinker, occasional drinker, and frequent drinker), five servings of vegetable and fruit (no/yes), exercise (none, 1–2 times/week, and >3 times/week), and depression symptoms were considered potential confounders of the multiple adjustment analysis. Depression symptoms were determined according to the respondent’s score on the Center for Epidemiology Studies Depression Scale (CES-D). Perceived stress was measured with a 10 item Perceived Stress Scale, which was the 4-item shortened version of the Perceived Stress Scale to assess stress in the past 2 weeks, using a 1–5 response scale [32]. Because of missing data of some of the items, score was calculated by averaging across the available items. Higher values reflected greater perceived stress. The Framingham Risk Score already takes into account age, sex, smoking, hypertension, HDL, and cholesterol; consequently, these variables were not added individually to the multiple adjusted models.

### Statistical analysis

A total of 1036 participants with available SEP data for at least of one time point were included in the current study. All estimates and standard errors were weighted to adjust for oversampling and unequal response rates by age, gender, and other covariates. Mean and standard deviation were presented for continuous variables with ANOVA tests for differences among the low, medium and high groups. Percentages were presented for categorical data and differences among the three SEP groups were tested by chi square analysis. In addition to the descriptive analysis of the participant by using SEP, we identified the distinct trajectories of life-course SEP; then used the trajectory group membership of each participant for subsequent analyses. Life-course SEP trajectory groups were determined using group-based trajectory modeling [33]. Analysis was implemented in PROC TRAJ of SAS software, which is a finite mixed modeling application that uses trajectory groups to statistically approximate unknown trajectories among population members [34]. The group-based trajectory approach we used involved fitting a polynomial model for each group. SEP of each group of participants over time is described using the binary logit distribution. The probability of staying in high SEP is directly measured through the proportion of individuals within each group staying in high SEP at a given time point. A total of 988 participants with available SEP data for four time points were classified into different trajectory groups according to the highest posterior probabilities of belonging to each group. The optimal number of groups was determined by comparing the Bayesian information criteria (BIC) and the principle of parsimony [35]. Multivariable linear regressions were then used to compare the levels of IL-6 and CRP of individuals in different trajectory groups.

## Results

Table 1 shows the characteristics of the SEBAS study population by childhood, young adulthood, active professional life, and older-age SEP. SEP at different time points of a life course were correlated. Percentages of low childhood SEP were higher among the low young adulthood SEP, low active professional SEP, and low older age SEP groups. Compared with participants of low or median SEP, those who were in the high SEP category had the lowest level of CRP and IL-6. Significant differences were observed among categories of young adulthood SEP, active professional life SEP, and older age SEP for CRP and between categories of young adulthood SEP and active professional life SEP for IL-6. There were no consistent and statistically discernible differences with regard to lifestyle factors such as smoking, alcohol consumption, having five servings of vegetable and fruit per day, or exercise across various SEP categories. The proportion of people having depressive symptoms were lower among the higher SEP categories.

**Table 1:** Characteristics of SEBAS study population by childhood, young adulthood, active professional and older-age SEP

Variable	Childhood SEP				Young adulthood SEP				Active professional life				Older age SEP			
*n*	Low	Med	High	p^a^	Low	Med	High	p	Low	Med	High	p	Low	Med	High	*p*
	466	168	398		268	381	380		180	446	410		456	307	237	
Social Demographic Variables																
Mean age, in year	66.7	66.7	63.1	<.001	71.1	64.1	62.5	<.001	71.8	64.8	62.9	<.001	65.7	65.1	63.7	0.03
(SD)	(9.1)	(10.5)	(9.3)		(8.8)	(8.8)	(9.1)		(8.6)	(9.0)	(9.2)		(9.2)	(9.4)	(9.8)	
Women, %	47.0	50.9	44.0	<.001	67.1	43.5	33.6	<.001	74.3	46.0	32.8	<.001	42.3	53.9	43.2	<.01
Low childhood SEP, %					75.3	53.0	19.4	<.001	76.4	56.7	21.4	<.001	55.0	47.1	31.5	<.001
Low young adulthood SEP, %	42.7	23,9	8.1	<.001					100	18.8	0.7	<.001	33.1	23.6	14.0	<.001
Low active professional SEP, %	29.4	15.0	5.3	<.001	68.0	0.0	0.0	<.001					21.3	16.1	10.0	<.001
Low older age SEP, %	53.8	46.6	36.5	<.001	59.4	53.0	29.6	<.001	59.5	47.2	44.3	<.001				
Physiological marker																
Hypertension: SBP > 140 mmHg or DBP > 90 mmHg or using anti-hypertensive medication, %	50.0	48.0	46.2	0.43	60.1	45.3	43.1	<.001	56.4	57.4	54.2	0.92	48.7	46.5	46.3	0.14
High total cholesterol: > = 240 mg/dL or using anti-hyperlipidemia medication, %	17.1	24.2	2.1	0.30	17.2	18.4	21.1	0.56	18.2	17.0	22.4	0.28	18.7	18.5	21.7	0.08
Low high-density lipoprotein (HDL) cholesterol: < 40 mg/dL, %	18.6	15.6	15.1	0.27	19.8	16.3	15.6	0.59	21.1	16.5	15.2	0.09	17.8	16.5	16.4	0.95
C-reactive protein (CRP, mg/dL), Mean	0.17	0.18	0.15	0.20	0.18	0.19	0.14	<.001	0.17	0.19	0.14	<.001	0.17	0.16	0.14	0.01
(SD)	(0.3)	(0.2)	(0.2)		(0.3)	(0.3)	(0.2)		(0.3)	(0.3)	(0.2)		(0.3)	(0.2)	(0.2)	
Interleukin-6 (IL-6, pg/mL), Mean	3.2	3.3	2.9	0.17	4.0	3.2	3.0	<.001	3.9	3.3	3.2	0.02	3.4	3.3	3.0	0.28
(SD)	(3.5)	(3.5)	(3.1)		(3.4)	(3.0)	(2.8)		(3.5)	(2.8)	(3.1)		(3.2)	(2.9)	(2.8)	
Behavioral Risk Factor																
Current smoker, %	20.6	16.6	18.5	0.49	13.5	23.1	19.3	<.01	8.5	23.0	20.0	<.01	22.8	16.0	16.8	0.05
Frequent or daily drinking,%	7.0	8.0	3.8	<.001	4.2	8.7	4.5	<.001	4.3	7.6	4.8	<.001	7.9	4.5	3.8	<.01
Less than five servings of vegetable and fruit per day, %	53.8	59.5	46.2	<.01	56.2	58.2	41.6	<.001	55.3	57.9	42.7	<.001	59.6	47.5	38.9	<.001
Exercise less than 3 times per week, %	55.4	53.9	54.1	<.01	63.7	55.9	46.3	<.001	65.0	57.0	46.6	<.001	62.6	50.4	41.4	<.001
Obesity(BMI > = 27)	26.4	27.6	23.3	0.09	27.6	28.4	20.6	0.03	25.9	28.7	21.2	0.05	25.0	26.6	24.0	0.17
Psychosocial Factor																
Depressive symptoms %	17.6	19.2	11.8	0.03	26.9	13.0	10.3	<.001	26.1	15.8	10.4	<.001	22.2	13.9	5.4	<.001
Perceived Stress (average score 0–4), Mean	3.0	2.9	2.0	0.20	2.9	3.0	3.0	0.08	2.9	3.0	3.0	0.13	2.9	3.0	3.2	<.001
(SD)	(0.6)	(0.7)	(0.6)		(0.7)	(0.6)	(0.6)		(0.7)	(0.6)	(0.6)		(0.7)	(0.7)	(0.5)	

a: *p*-value of ANOVA test for continuous variables and *p*-value of chi-square test for categorical variables

**Fig. 1:**
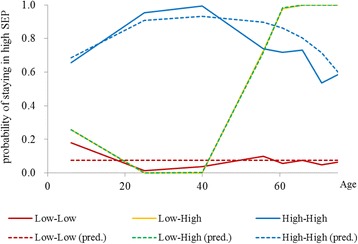
Group-based trajectories of life-course SEP. BIC = −2094.78 (3900 observations); BIC = −2088.60 (988 persons). Group 1, Low-Low (36.5%); Group 2, Low-High (26.8%); Group 3, High-High (36.7%)

**Fig. 2:**
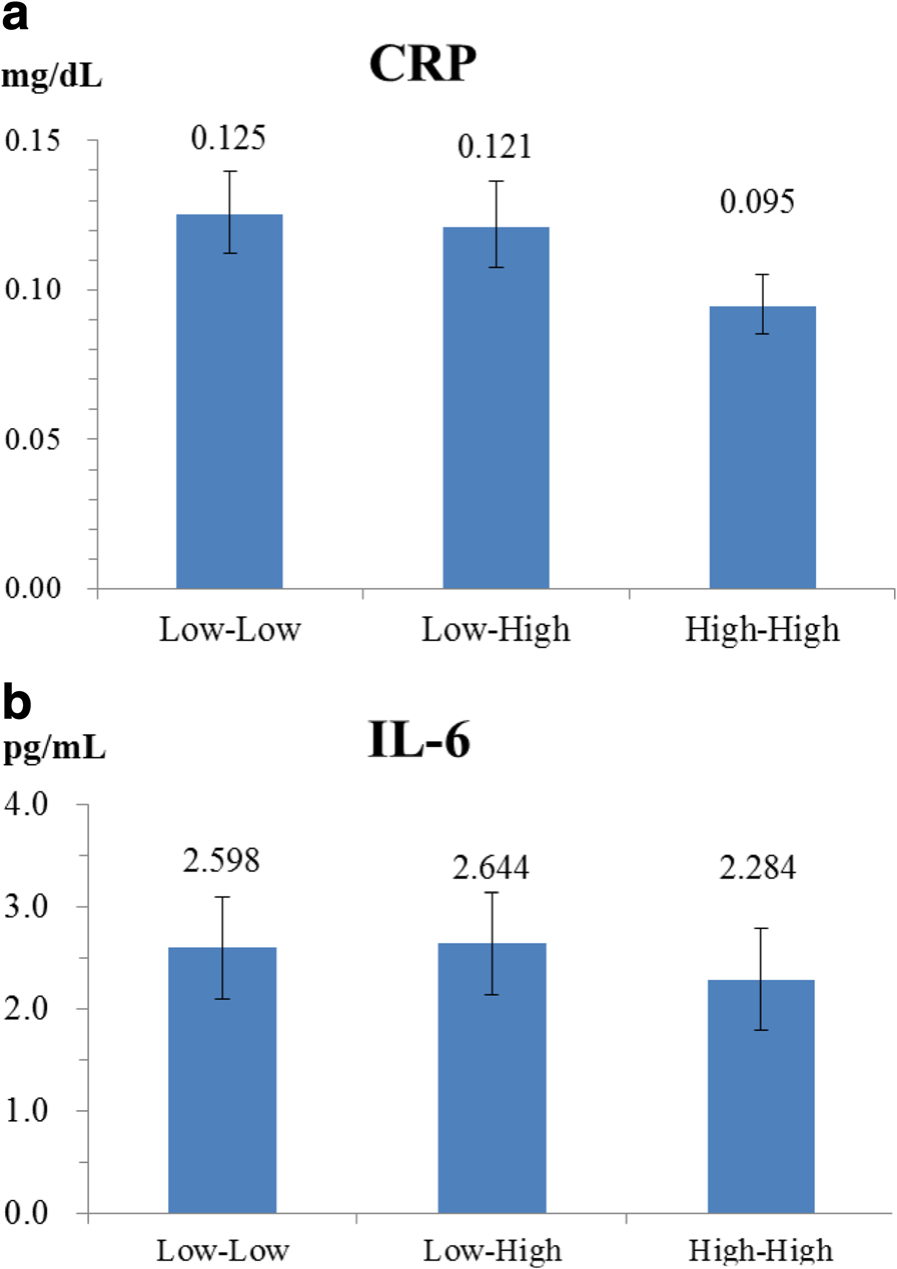
Multiple adjusted mean concentrations of CRP and IL-6 by groups of life-course SEP Covariates adjusted for the estimation of mean concentrations of life-course SEP include disease, BMI, Framingham risk score, health behavior (alcohol drinking, diet, and exercise), and depression. a CRP. b IL-6

Three distinct SEP trajectory groups were determined to describe the pattern of social mobility that the participants were involved in through their life span from family of origin to adulthood and older age (Fig. 1). Group 1 (Low-Low) represented 36.5% of the participants who remained in a relatively lower SEP among the four time points; Group 2 (Low-High) comprised 26.8% of the participants who showed an upward mobility of life-course SEP from childhood to later in life; Group 3 consisted of the remaining 36.7% of participants who stayed in a relatively higher SEP throughout their life course. The results showed a social gradient in level of inflammation that runs from top to bottom of the SEP trajectories. A descending trend in levels of multiple adjusted CRP from 0.125 mg/dL [95% confidence interval (CI): 0.112–0.140 mg/dL] and 0.121 mg/dL (95% CI: 0.108–0.136 mg/dL) to 0.095 mg/dL (95% CI: 0.085–0.105 mg/dL) was observed from Low-Low and Low-High to High-High. The levels of IL-6 were 2.598 pg/mL (95% CI: 2.410–2.801 pg/mL), 2.644 pg/mL (95% CI: 2.436–2.869 pg/mL), and 2.284 pg/mL (95% CI: 2.125–2.454 pg/mL) for the Low-Low, Low-High, and High-High groups, respectively. No obvious increasing or decreasing trends were observed (Fig. 2). The participants in the High-High group had the lowest levels of CRP and IL-6. Compared with those in the Low-Low group, the participants in the Low-High group had a similar adjusted CRP (−0.032 ln mg/L; 95% CI: −0.193, 0.128); participants in the High-High group had a significantly lower level of adjusted CRP concentration (−0.279 ln mg/L; 95% CI: −0.434, −0.125). Likewise, the participants in the High-High group had a significantly lower level of adjusted IL-6 concentration (−0.129 ln pg/mL; 95% CI −0.236, −0.023). The coefficients attenuated but remained nearly the same after we controlled for gender, age, BMI, established risk factors, depressive symptoms, and stress. (Table 2).

**Table 2:** Multivariable linear regression of life-course SEP groups

	CRP(ln mg/dL)						IL-6 (ln pg/mL)					
	SEP (Ref: Low-Low)						SEP (Ref: Low-Low)					
	Low-high			High-High			Low-High			High-High		
	*β*	(95% CI)	p	*β*	(95% CI)	p	*β*	(95% CI)	p	*β*	(95% CI)	p
Model 1	−0.059	(−0.219, 0.102)	0.47	**−0.289**	**(−0.438, −0.139)**	**<.001**	−0.025	(−0.136, 0.086)	0.66	**−0.149**	**(−0.252, −0.045)**	**0.005**
Model 2	−0.060	(−0.219, 0.100)	0.46	**−0.273**	**(−0.422, −0.124)**	**<.001**	−0.017	(−0.127, 0.093)	0.76	**−0.136**	**(−0.238, −0.033)**	**0.010**
Model 3	−0.052	(−0.209, 0.105)	0.52	**−0.295**	**(−0.441, −0.149)**	**<.001**	0.002	(−0.106, 0.111)	0.97	**−0.149**	**(−0.250, −0.048)**	**0.004**
Model 4	−0.038	(−0.196, 0.120)	0.63	**−0.286**	**(−0.438, −0.134)**	**<.001**	0.009	(−0.100, 0.118)	0.88	**−0.138**	**(−0.243, −0.033)**	**0.010**
Model 5	−0.022	(−0.181, 0.137)	0.79	**−0.276**	**(−0.430, −0.122)**	**<.001**	0.018	(−0.091, 0.128)	0.75	**−0.127**	**(−0.233, −0.021)**	**0.019**
Model 6	−0.032	(−0.193, 0.128)	0.69	**−0.279**	**(−0.434, −0.125)**	**<.001**	0.017	(−0.093, 0.128)	0.76	**−0.129**	**(−0.236, −0.023)**	**0.018**
Model 7	−0.044	(−0.186, 0.098)	0.54	**−0.193**	**(−0.330, −0.056)**	**0.006**	0.028	(−0.070, 0.126)	0.58	−0.040	(−0.135, 0.055)	0.41

Values of CRP and IL-6 were log transformed to adjust for their skewed and wide distributions

Bold values denote where the 95% confidence intervals (95% CIs) encompass the reference category

Model 1: Adjusted for age, gender, and disease

Model 2: Adjusted for covariates in Model 1 and BMI

Model 3: Adjusted for covariates in Model 2 and Framingham risk score

Model 4: Adjusted for covariates in Model 3 and health behavior (alcohol drinking, diet, and exercise)

Model 5: Adjusted for covariates in Model 4 and depression

Model 6: Adjusted for covariates in Model 5 and stress

Model 7: Adjusted for covariates in Model 6 and the other inflammatory marker

## Discussion

We found three distinct life-course SEP groups by using the time points of childhood, young adulthood, active professional life and older age. Rapid industrialization and economic growth in Taiwan was recorded during the latter half of the twentieth century [36]. Higher education has expanded rapidly since the 1960s [37]. Industrialization and economic growth would have resulted in improvement in education and occupation status of the whole population and might explain why trajectories across four time points fit into only three groups and why a “high-low” group was not classified.

Low SEP in childhood was associated with elevated CRP and IL-6 in older age. Similar to those who remained in lower SEP among the four time points, the respondents who transited from low SEP in childhood and young adulthood to high SEP in active professional life had higher CRP and IL-6 in older age. Although the relationships between SEP and IL-6 levels were nonsignificant after adjustment for CRP, directionality of the relationships was observed.

Previous observational studies have demonstrated that low SEP was associated with higher inflammatory biomarkers. In a cross-sectional study on 2729 middle-aged participants from the U.S. Framingham Offspring Study cohort, the number of years of education was inversely associated with CRP (beta for logarithmic CRP: −0.0034, *p* < 0.001) and IL-6 (beta for logarithmic IL-6: −0.011, *p* < 0.07) levels after adjustment for biological and psychosocial confounders [9]. The findings of the current study are similar, suggesting that only CRP had a significant association, but not IL-6. In another cross-sectional study on 8998 hospital-based, healthy, and elite executive participants, a significant inverse association between the number of school years and CRP was reported. After adjustment for multiple potential confounders, the percentages of mean change in CRP levels for each additional school year were −2.3% (95 CI: −3.2% to −1.3%) for men and −2.0% (95 CI: −3.1% to −0.8%) for women [38]. Our findings are compatible with the aforementioned studies. Participants who remained in a high SEP throughout their life course had the lowest levels of CRP and IL-6 concentrations.

Several studies have reported associations between adverse childhood experiences and chronic or age-related conditions. However, no consensus is reached in the literature regarding whether childhood SEP or recent SEP is more strongly related to inflammatory biomarkers. In a cohort study comprising 12,681 Caucasian and African American people, cumulative exposure to lower SEP throughout a life course and low adult SEP were associated with elevated CRP levels [39]. Furthermore, among the 5951 respondents of the British birth cohort study, significantly inverse associations were observed for childhood and early adulthood but not for SEP in older age [40]. A follow up study of the British birth cohort suggested that childhood socioeconomic position acts either as a sensitive period or as part of a cumulative life course model [13]. Our data support that early childhood SEP was a critical period and had stronger effects than those of other periods. The upward social mobility in adulthood could be attributed to the occupational prestige gained from active professional life and may have only marginal effects on inflammation in older age.

Health behavior and psychological distress were reported to be the two major pathways by which SEP may influence the levels of inflammatory biomarkers [41, 42]. An adverse childhood experience predicts an elevated risk of depression, a clustering of metabolic risk factors, and elevated inflammation levels in adult life. In a cross-sectional study on 2266 randomly selected samples in Greece, those who reported “high” education had 45% lower CRP, but the association was mainly explained by the adoption of an unhealthy lifestyle, such as increased smoking habits, physical inactivity, and obesity [43]. In a study on 851 men and women aged 30–54 years, SEP was no longer associated with IL-6 after adjustment for lifestyle factors [8]. The results of the current study show no consistent and statistically discernible differences with regard to lifestyle factors such as smoking, alcohol consumption, having five servings of vegetable and fruit per day, or exercise across various SEP categories. Higher SEP was associated with lower percentages of depressive symptoms. The associations between life-course SEP and CRP or IL-6 were attenuated but remained significant after we controlled for gender, age, BMI, established risk factors, depressive symptoms, and stress. The results are consistent with the findings of the 1958 British birth cohort study, showing that controlling for BMI, smoking, and physical activity had little effect on the associations between SEP and CRP [40].

CRP is a marker of systemic inflammation, and IL-6 acts as both a pro-inflammatory and anti-inflammatory cytokine in mediating inflammation. Levels of IL-6 and CRP are physiologically linked because of the function of IL-6 on hepatic synthesis and the excretion of CRP. Markers of systemic inflammation, pro-inflammatory cytokines and anti-inflammatory cytokines may reflect different features of atherosclerotic processes and the interplay between life-course SEP and inflammation; thus, both CRP and IL-6 were associated with life-course SEP after adjustment for the covariates. The biological mechanisms through which SEP exerts its effects on inflammation is limited. Low SEP across the life-course was reported to be associated with increased inflammatory activity, and may have an impact on health via inflammation through the interconnection of lifestyle factors and genetic regulation of immune function. A study on Italian participants of a large scale prospective cohort in Europe demonstrated that indicators of social economic status were associated with DNA methylation of genes involved in inflammation and suggested that social adversity represented by low economic status might leave an epigenetic mark on cells, which would exacerbate the inflammatory response [24].

The strengths of this study are as follows: First, we used semi-longitudinal data based on subjective recall from a nationally representative cohort to provide an enhanced opportunity to examine how life-course SEP predicts inflammation in older age. Second, we use repeated measurement of SEP at four time points throughout a life course. Third, in contrast to other studies observing the overall pattern among the available time points, we used group-based trajectory modeling to identify distinct social mobility groups of SEP throughout a life course. This novel statistical approach provides clearer insight into hypothesis testing within the proposed frameworks. In addition to the subjective measure of SEP with education and occupation, we used an objective measure of SEP that has been demonstrated to be a significant predictor of health, even in the presence of conventional measures of SEP for older Taiwanese people [30]. Nevertheless, the limitations of this study must be considered in interpreting the findings. First, the inflammatory markers and covariates were measured at a single time point and may not accurately capture the time-varying nature of the variables. Second, only the father’s education and occupation were collected for the cohort study. Admittedly, we were not able to consider the mother’s education and occupation for the analysis. However, during the participants’ childhood, not many women in Taiwan’s patriarchal society were provided with education, and few of them had jobs. Therefore, the fathers’ education and occupation were more practical choices for measuring the participants’ SEP in childhood. In addition, data collection on infectious illnesses and conditions associated with elevated inflammatory markers was limited in this study and may have resulted in residual confounding due to the systemic nature of CRP and IL-6*.* No hard endpoints, such as cardiovascular morbidity or deaths, were analyzed.

The main focus of this study was to identify the possible upward, downward or stable trajectories for later modelling. The identification of trajectory groups is an inductive approach that allows the data to ‘speak for themselves’ by identifying groups of individuals whose SEP follows a similar course over time, highlighting differences and similarities between these individuals which call for explanation, and providing a foundation on which later modelling can be based. However, due to the constraints of limited sample size, we could just dichotomize the four SEP variables at different life stages.

## Conclusions

In conclusion, life-course SEP is related to inflammation in older age. Low SEP in childhood is associated with elevated inflammatory markers in older age. Even after transit from low SEP in childhood to high SEP in older age, the risk remains. This study demonstrates the social inequalities in population health. The health of people who remained stable in an advantaged SEP is better when compared to that of those who remained in the disadvantaged SEP or those who upwardly moved from a disadvantaged position to an advantaged position. Our data support the notion that childhood SEP may act either as a sensitive period or as part of the accumulation of risk. Intervention in early childhood and strategies for ensuring that every child has an optimal start in life are crucial for reducing the burdens of inflammation.

## Abbreviations

BAS: Below assay sensitivity
BMI: Body mass index
CES-D: Center for Epidemiology Studies Depression Scale (CES-D)
CI: Confidence interval
CRP: C-reactive protein
CV: Coefficient of variation
DBP: Diastolic blood pressure
HDL: High-density lipoprotein cholesterol
IL-6: Interleukin-6
IQR: Interquartile range
p75: The 75th percentile
SBP: Systolic blood pressure
SD: Standard deviation
SEBAS: Social Environment and Biomarkers of Aging Study
SEP: Socioeconomic position
TLSA: Taiwan Longitudinal Survey of Aging
